# Identification of RFX5 as prognostic biomarker and associated with immune infiltration in stomach adenocarcinoma

**DOI:** 10.1186/s40001-022-00794-w

**Published:** 2022-08-31

**Authors:** Lili Guo, Dingsheng Liu

**Affiliations:** 1 Department of Anesthesiology, The First Hospital of China Medical University, Shenyang, China; 2 Department of General Surgery, Shengjing Hospital of China Medical University, Shenyang, 110004 China

**Keywords:** Stomach adenocarcinoma, Prognosis, RFXs, Immune infiltration

## Abstract

**Background:**

Regulatory factor X (RFX) gene family is a series of encodes transcription factors with a highly conserved DNA binding domain. RFXs played a vital role in the development and progression of cancer. However, the significance of RFXs in stomach adenocarcinoma (STAD) has not been fully clarified.

**Methods:**

Online bioinformatics tools such as GSCALite, Kaplan–Meier Plotter, TIMER, LinkedOmics were used to explore the immunomodulatory function and clinical value of RFXs in STAD.

**Results:**

The mRNA level of RFX1, RFX3, RFX4, RFX5, RFX7 and RFX8 was significantly elevated in STAD tissue versus adjacent normal tissue. We also summarize the copy number variation, single nucleotide variants and drug sensitivity of RFXs in STAD. Prognostic analysis indicated that STAD patients with high RFX5 and RFX7 expression had a better overall survival, first progression, and post-progression survival. Moreover, RFX5 expression was significantly associated with the abundance of immune cells, the expression of immune biomarkers and tumor mutational burden score in STAD. Functional enrichment analysis revealed that RFX5 and its related genes were mainly involved in T cell activation, antigen receptor-mediated signaling pathway, cell adhesion molecules, and Th17 cell differentiation. Validation study further verified the expression and prognosis of RFX5 in STAD. Further univariate and multivariate analyses suggested that pathological stage and RFX5 could be a potential independent prognostic factor for STAD.

**Conclusions:**

RFX5 was a candidate prognostic biomarker and associated with immune infiltration in STAD.

**Supplementary Information:**

The online version contains supplementary material available at 10.1186/s40001-022-00794-w.

## Introduction

Gastric cancer is the fifth most common and the third most lethal cancer worldwide [1]. Stomach adenocarcinoma (STAD) ranked more than 95% of gastric cancer cases. Moreover, great progresses have been made in the treatment of STAD, including surgery, chemoradiotherapy and immunotherapy [2, 3]. However, the prognosis for patients with advanced STAD remains poor, with a 5-year survival rate of less than 30% [4]. Moreover, the overall survival rate for STAD patients with advanced or metastatic disease is less than 1 year [5]. Despite some risk factors had been identified for STAD, including Helicobacter pylori infection and high salt intake [4], the molecular mechanism of STAD was not still fully clarified. Thus, it is vital to explore some important genes related to the occurrence and development of STAD and identify the mechanism and prognostic biomarkers for STAD.

Regulatory factor X (RFX) gene family is a series of encodes transcription factors with a highly conserved DNA binding domain [6]. A total of 8 members of RFX gene family (RFX1-8) have been identified in mammal. Previous study revealed that RFX played a vital role in the development and progression of cancer. Hang et al. suggested that high expression of RFX4 was related to tumor progression and poor prognosis in glioblastoma [7]. Moreover, RFX1 could induce down-regulation of transforming growth factor β2 transcription in neuroblastoma [8]. However, the expression, prognostic value and other vital role of RFX gene family in STAD had not been fully clarified.

In our work, data mining was performed to clarify the expression level, prognostic value, and potential mechanisms of RFX gene family in STAD. Moreover, we also verified our result by performing vitro experiments. Our result may provide more evidence about the prognostic biomarker of STAD.

## Materials and methods

### Expression analysis of RFXs in STAD

After downloading the transcriptomic data of 415 STAD from TCGA database on March 8, 2022, we normalize the data to transcripts per million reads (TPM). Student's t-test was performed to explore the difference of RFXs between STAD and normal gastric tissues. Kruskal–Wallis test was performed to evaluate the differences of RFXs in different stage of STAD patients with *p* < 0.05 as the threshold value.

### GSCALite

The GSCALite database (http://bioinfo.life.hust.edu.cn/web/GSCALite/) is a TCGA database visualization platform for the analyzation of copy number variation (CNV), single nucleotide variants (SNV) and drug sensitivity [9]. In our study, the genetic mutation and drug sensitivity of RFXs in STAD was explored using GSCALite. Pearson correlation analysis was performed to analyze the RFXs expression and drug sensitivity.

### Kaplan–Meier Plotter

The Kaplan–Meier Plotter (http://www.kmplot.com/) is a TCGA database visualization platform for the prognostic analysis [10]. Prognosis analyses [overall survival (overall survival, OS), first progression (FP), and post-progression survival (PPS)] of RFXs in STAD were performed with Kaplan–Meier Plotter, with *p* < 0.05 indicating statistical significance.

### Immune infiltration analysis

After obtaining the level of immune cells and immune-related biomarkers of TIMER (cistrome.shinyapps.io/timer) database, an online database for tumor microenvironment analysis [11]. The R software “ggstatsplot” package was used to draw the correlations between RFXs expression and immune score. In addition, the relationship between somatic cell copy number variation of RFXs and immune infiltration was further explored through the “SCNA” module in TIMER. Spearman correlation analysis was performed to evaluate the correlation between RFXs expression and immune cell biomarkers in STAD. Differences were considered statistically significant at *p* < 0. 05.

### Functional enrichment analysis

We first obtained RFXs-correlated genes using the LinkedOmics (http://www.linkedomics.org/), a TCGA database visualization platform can provide various analyses such as gene expression correlation analysis [12]. We then performed GO and KEGG pathway analysis based on these RFXs-correlated genes using R package “ggplot2” with enrichment *p* < 0.05. In addition, RFXs-related miRNA targets and transcription factors (TFs) were explored with LinkedOmics in LinkFinder module.

### Validation of the expression and prognosis value of RFXs in STAD

The protein level of RFXs STAD was verified using The Human Protein Atlas (https://www.proteinatlas.org/), a tissue-based map of the human proteome [13]. After obtaining 60 cases of STAD and paired normal tissues, we used TRIzol kit (Vazyme, Nanjing, China) to extract total RNA from STAD tissues and normal gastric tissues. RT-qPCR experiments were used to verify RFXs in STAD. RFX5 primers were designed using the NCBI website with upstream sequence: 5ʹ-3ʹ GATGAGCCTGATGCTAAGAGC and downstream sequence 5ʹ-3ʹ CCCTCTACTTTGTTCTGCACG. The primers of internal reference GAPDH primers were: upstream sequence 5ʹ-3ʹ CCACATCGCTCAGACACCAT and downstream sequence 5ʹ-3ʹ GGCAACAATATCCACTTTACCAGAGT.

## Results

### The expression level of RFXs in STAD

**Fig. 1:**
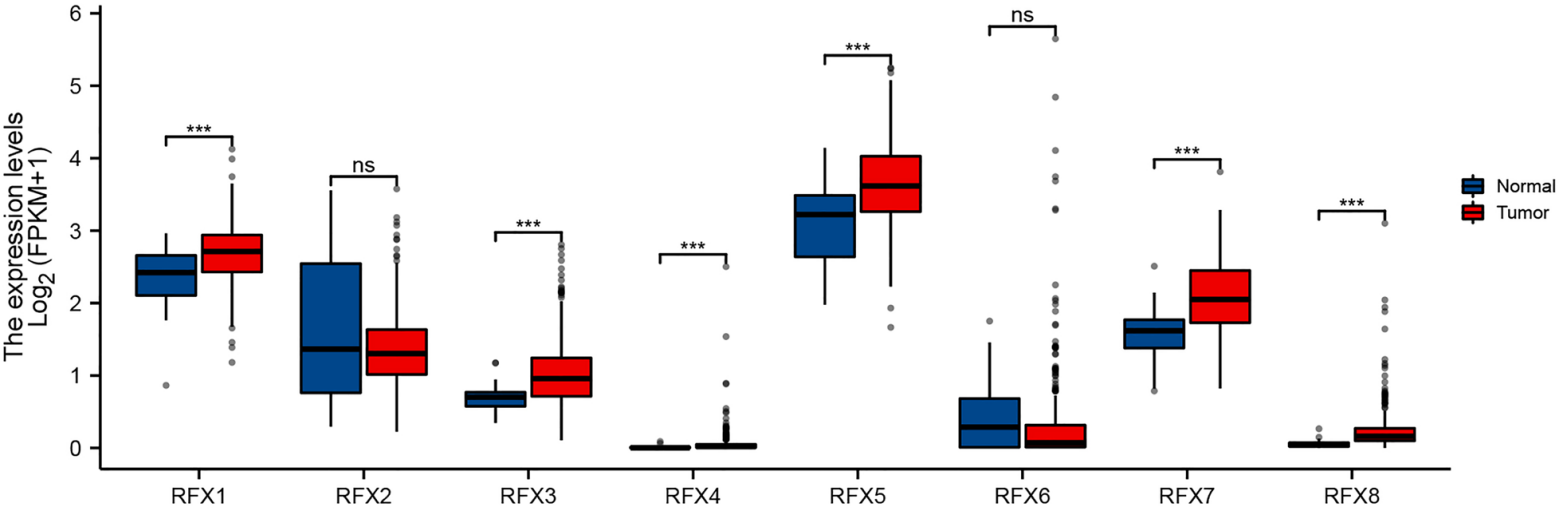
The mRNA levels of RFXs in STAD. The mRNA level of RFX1, RFX3, RFX4, RFX5, RFX7 and RFX8 was significantly elevated in STAD tissue versus adjacent normal tissue. STAD, stomach adenocarcinoma. ***p < 0.001

**Fig. 2:**
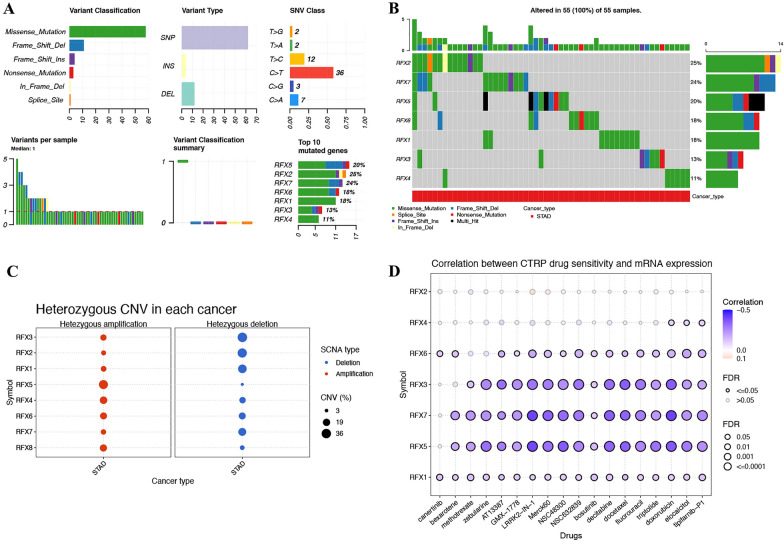
Genetic mutation and drug sensitivity of RFXs in STAD. (**A**-**B**) Single nucleotide variants of RFXs in STAD. (**C**) Copy number variation of RFXs in STAD. (**D**) Spearman's correlation analysis of the sensitivity of RFXs to drugs. STAD, stomach adenocarcinoma

The expression oncoplot of RFXs is shown in Fig. 1, revealing that the mRNA level of RFX1, RFX3, RFX4, RFX5, RFX7 and RFX8 was significantly elevated in STAD tissue versus adjacent normal tissue (all *p* < 0.001). However, there is no significant difference of RFXs among STAD patients in stage I–IV (Additional file 1: Fig. S1). Figure 2A–C shows genetic mutation oncoplot of RFXs in STAD. Among these RFXs, the top three genes with the highest variation rates were RFX2 (25%), RFX7 (24%), and RFX5 (20%) (Fig. 2A, B). Genetic variation analysis revealed that missense mutation was the most common variant classification and C > T was the most common SNV class (Fig. 2A). CNV analysis revealed that RFX5/8 had copy number amplification while the CNV deletion frequency of RFX1/2/3/7 was widespread (Fig. 2C). The main reasons for STAD treatment failure are the presence of drug resistance. In our study, drug sensitivity revealed that low expression of RFX3/5/7 was resistant to most drugs (Fig. 2D). Combined with these results, we selected RFX5 and RFX7 for further study.

### The prognostic significance of RFX5/7 in STAD

**Fig. 3:**
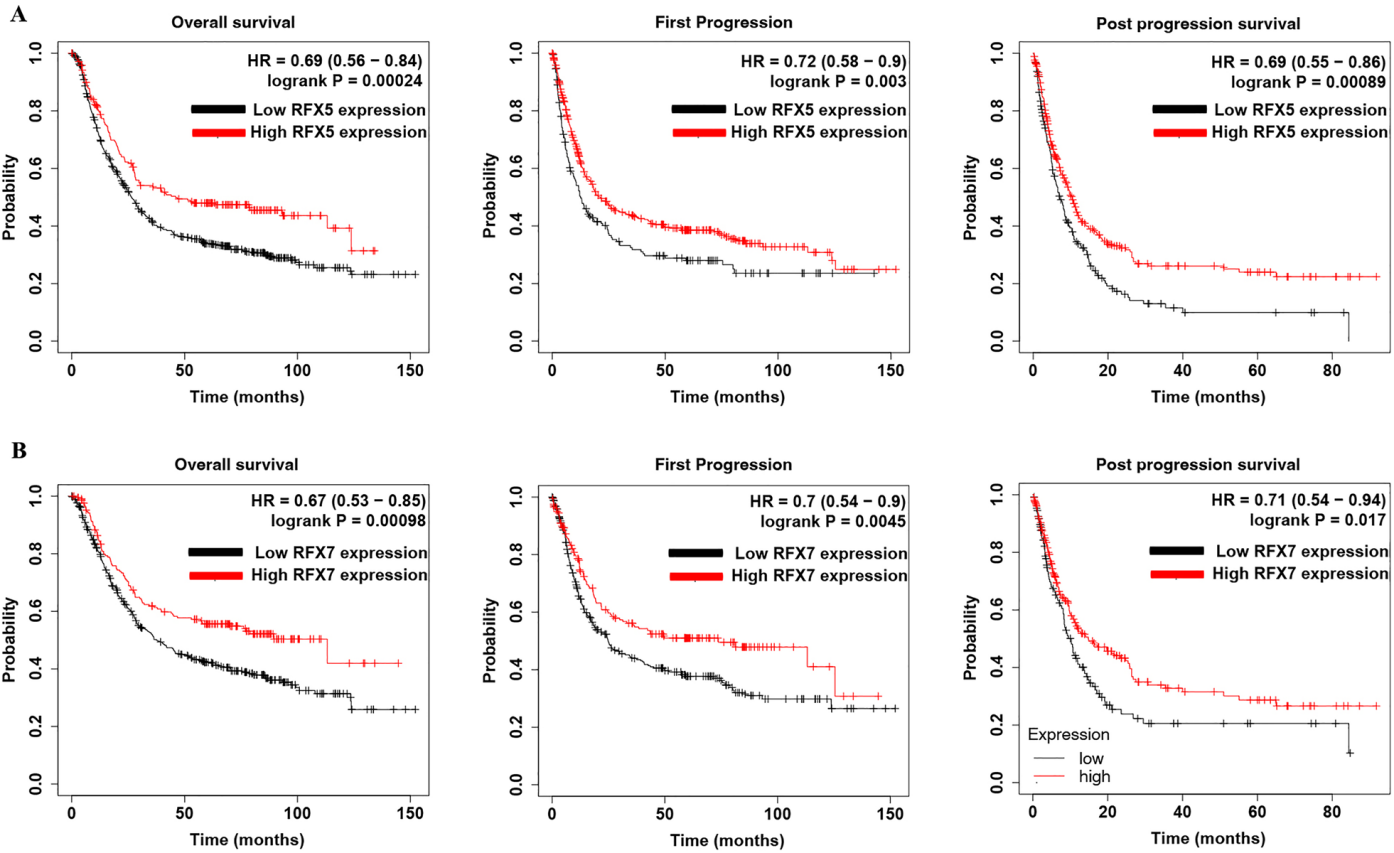
Prognostic significance of RFXs in STAD. **A** STAD patients with high RFX5 expression had a better OS, FP, and PPS in STAD versus those with low RFX5 expression. **B** STAD patients with high RFX7 expression had a better OS, FP, and PPS in STAD versus those with low RFX7 expression. OS, overall survival; FP, first progression; PPS, post-progression survival. STAD, stomach adenocarcinoma

As shown in Fig. 3A, STAD patients with high RFX5 expression had a better OS (*p* = 0.00024, HR = 0.69), FP (*p* = 0.003, HR = 0.72), and PPS (*p* = 0.00089, HR = 0.69) in STAD versus those with low RFX5 expression. Moreover, STAD patients with high RFX7 expression had a better OS (*p* = 0.00098, HR = 0.67), FP (*p* = 0.0045, HR = 0.7), and PPS (*p* = 0.017, HR = 0.61) in STAD versus those with low RFX7 expression (Fig. 3B). These data suggested RFX5 and RFX7 as potential prognosis biomarkers for STAD.

### RFX5 was correlated with immune infiltration in STAD

**Fig. 4:**
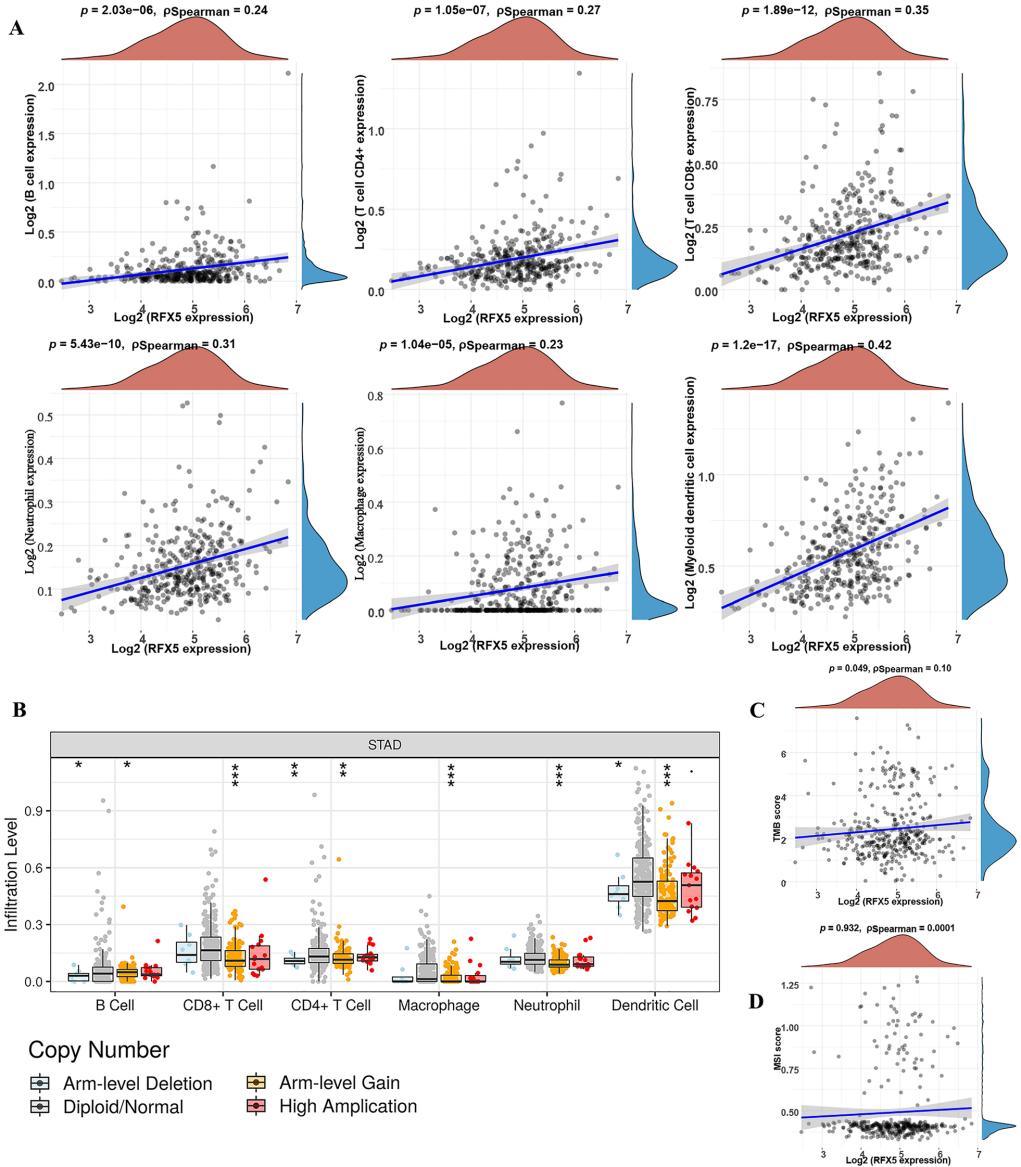
The correlation of RFX5 and immune cell infiltration in STAD. **A** Significant positive correlation between RFX5 level and B cells, CD4 + T cells, CD8 + T cells, neutrophils, macrophages, and myeloid dendritic cells. **B** Correlation between RFX5 SCNA and immune cell infiltration. **C**, **D** Correlation between RFX5 level and TMB score/MSI score. SCNA, somatic copy number alterations; TMB, tumor mutational burden; MSI, microsatellite instability. *p < 0.05; **p < 0.01; ***p < 0.001

Among RFX5 and RFX7, we selected RFX5 for further analysis. We then analyzed the correlation between immune cell infiltration and RFX5 expression. As expected, RFX5 expression was significantly positively correlated with the abundance of B cells (*p* = 2.03e−6), CD4 + T cells (*p* = 1.05e−7), CD8 + T cells (*p* = 1.89e−12), neutrophils (*p* = 5.43e−10), macrophages (*p* = 1.04e−5) and myeloid dendritic cells (*p* = 1.2e−17) (Fig. 4A). We also found that CNV of RFX5 could partly suppress immune cell infiltration (Fig. 4B, all *p* < 0.05). Further analysis revealed that RFX5 expression was significantly correlated with TMB score (Fig. 4C, *p* = 0.049) but not MSI score (Fig. 4D, *p* = 0.932) of STAD. We further explored the correlation between RFX5 expression and immune-related biomarkers. The result is shown in Table 1. Interestingly, RFX5 expression was significantly positively correlated with the expression of the biomarkers of CD8 + T cell (CD8A, CD8B) T cell (CD3D, CD3E, CD2), B cells (CD19, CD79A), monocyte (CD86, CSF1R), M2 macrophage (CD163, VSIG4, MS4A4A), dendritic cell (HLA-DPB1, HLA-DQB1, HLA-DRA, HLA-DPA1, CD1C, NRP1, ITGAX), Th1 cell (TBX21, STAT4, STAT1, IFNG, TNF), Th2 cell (GATA3, STAT6, STAT5A, IL13), Tfh (BCL6, IL21), Treg (FOXP3, CCR8, STAT5B, TGFB1) and T cell exhaustion (PDCD1, CTLA4, LAG3, HAVCR2, GZMB). These evidences revealed that RFX5 may play a vital role in the tumor microenvironment of STAD.

**Table 1:** The correlation between RFX5 and immune-related biomarkers in STAD

Description	Biomarkers	GEPIA		TIMER	
		Cor	*P*-value	Cor	*P*-value
CD8 + T cell	CD8A	0.39	^c^	0.437	^c^
	CD8B	0.15	^b^	0.304	^c^
T cell (general)	CD3D	0.34	^c^	0.347	^c^
	CD3E	0.32	^c^	0.394	^c^
	CD2	0.38	^c^	0.407	^c^
B cell	CD19	0.25	^c^	0.314	^c^
	CD79A	0.23	^c^	0.24	^c^
Monocyte	CD86	0.3	^c^	0.354	^c^
	CSF1R	0.34	^c^	0.377	^c^
TAM	CCL2	0.017	0.73	0.114	^a^
	CD68	0.22	^c^	0.22	^c^
	IL10	0.091	0.065	0.375	^c^
M1 macrophage	NOS2	0.038	0.45	0.028	0.585
	IRF5	0.27	^c^	0.282	^c^
	PTGS2	0.039	0.43	− 0.043	0.399
M2 macrophage	CD163	0.29	^c^	0.417	^c^
	VSIG4	0.2	^c^	0.289	^c^
	MS4A4A	0.27	^c^	0.353	^c^
Neutrophils	CEACAM8	0.059	0.24	− 0.034	0.514
	ITGAM	0.35	^c^	0.423	^c^
	CCR7	0.21	^c^	0.336	^c^
Natural killer cell	KIR2DL1	0.11	^a^	0.173	^c^
	KIR2DL3	0.23	^c^	0.101	0.0502
	KIR2DL4	0.23	^c^	0.233	^c^
	KIR3DL1	0.14	^b^	0.165	^b^
	KIR3DL2	0.28	^c^	0.289	^c^
	KIR3DL3	0.031	0.53	0.107	^a^
	KIR2DS4	0.16	^c^	0.156	^b^
Dendritic cell	HLA-DPB1	0.476	^c^	0.509	^c^
	HLA-DQB1	0.22	^c^	0.387	^c^
	HLA-DRA	0.55	^c^	0.531	^c^
	HLA-DPA1	0.51	^c^	0.507	^c^
	CD1C	0.14	^b^	0.181	^c^
	NRP1	0.21	^c^	0.266	^c^
	ITGAX	0.34	^c^	0.403	^c^
Th1	TBX21	0.43	^c^	0.456	^c^
	STAT4	0.35	^c^	0.42	^c^
	STAT1	0.53	^c^	0.47	^c^
	IFNG	0.21	^c^	0.356	^c^
	TNF	0.19	^c^	0.194	^c^
Th2	GATA3	0.2	^c^	0.285	^c^
	STAT6	0.28	^c^	0.362	^c^
	STAT5A	0.55	^c^	0.526	^c^
	IL13	0.02	0.69	0.131	^c^
Tfh	BCL6	0.31	^c^	0.28	^c^
	IL21	0.34	^c^	0.288	^c^
Th17	STAT3	0.41	^c^	0.431	^c^
	IL17A	− 0.034	0.5	0.012	0.821
Treg	FOXP3	0.39	^c^	0.459	^c^
	CCR8	0.46	^c^	0.422	^c^
	STAT5B	0.37	^c^	0.404	^c^
	TGFB1	0.19	^c^	0.196	^c^
T cell exhaustion	PDCD1	0.41	^c^	0.511	^c^
	CTLA4	0.2	^c^	0.488	^c^
	LAG3	0.32	^c^	0.399	^c^
	HAVCR2	0.43	^c^	0.394	^c^
	GZMB	0.18	^c^	0.604	^c^

^a^*p*-value < 0.05

^b^*p*-value < 0.01

^c^*p*-value < 0.001

### Functional enrichment analysis

**Fig. 5:**
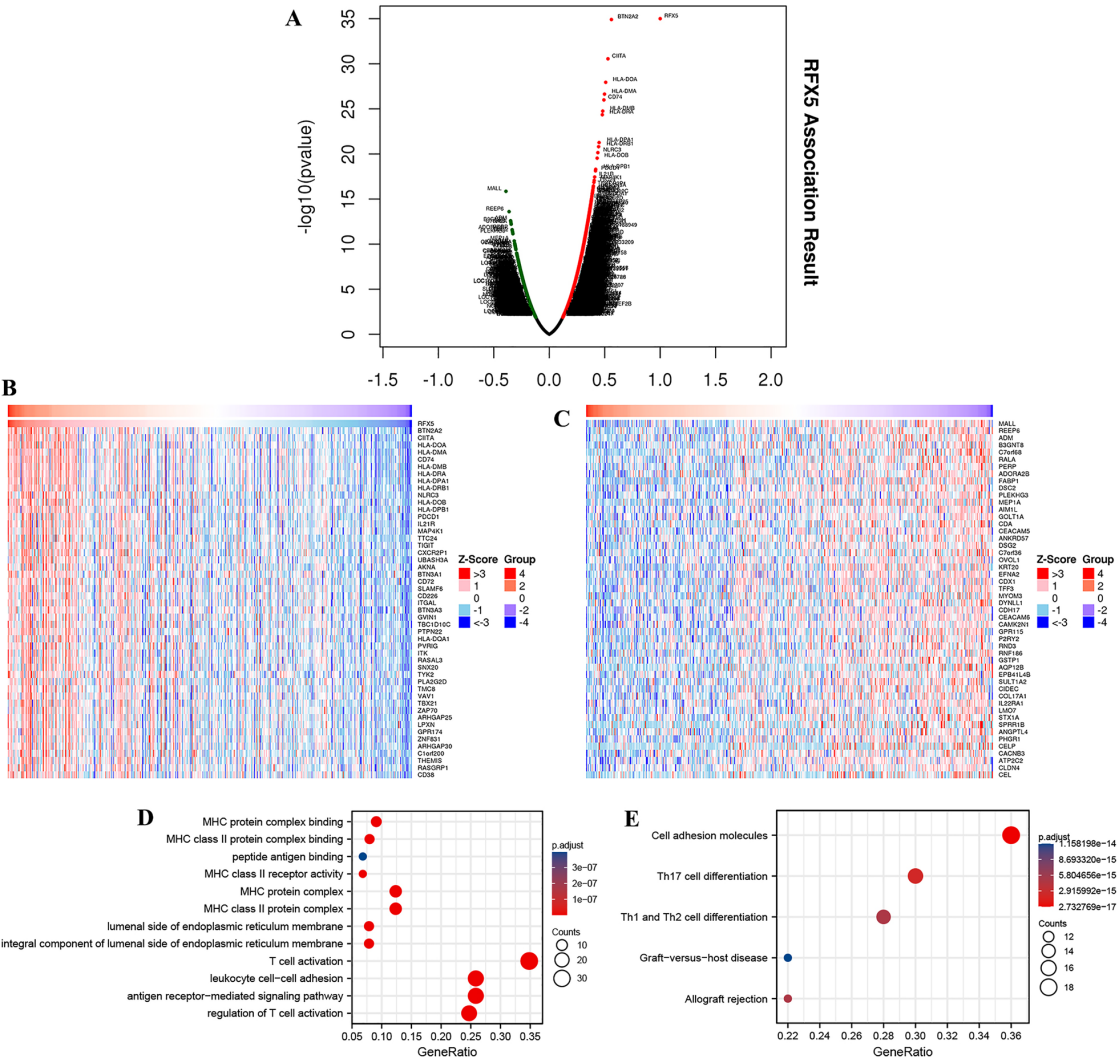
Interacting genes of RFX5 and functional enrichment analysis. **A** Volcano plot of interacting genes of RFX5 in STAD. Red indicates positively related genes, and green indicates negatively related genes. **B**, **C** Heatmaps revealed the TOP 50 genes positively and negatively correlated with RFX5 in STAD. **D**, **E** The enriched items of GO and KEGG analysis

Volcano in Fig. 5A reveals RFX5-correlated genes. As a result, a total of 7398 genes were obtained. The top 50 genes that were most positively and negatively associated with RFX5 are shown in Fig. 5B and C, respectively, (*P* < 0.05). Functional enrichment analysis revealed that RFX5 and correlated genes were correlated with MHC protein complex binding, T cell activation, leukocyte cell–cell adhesion, and antigen receptor-mediated signaling pathway (Fig. 5D). Moreover, The KEGG pathway analysis demonstrated the involvement of FX5 and correlated genes in cell adhesion molecules, Th17 cell differentiation, and Th1/2 cell differentiation (Fig. 5E).

### RFX5-associated transcription factor and miRNA target in STAD

**Fig. 6:**
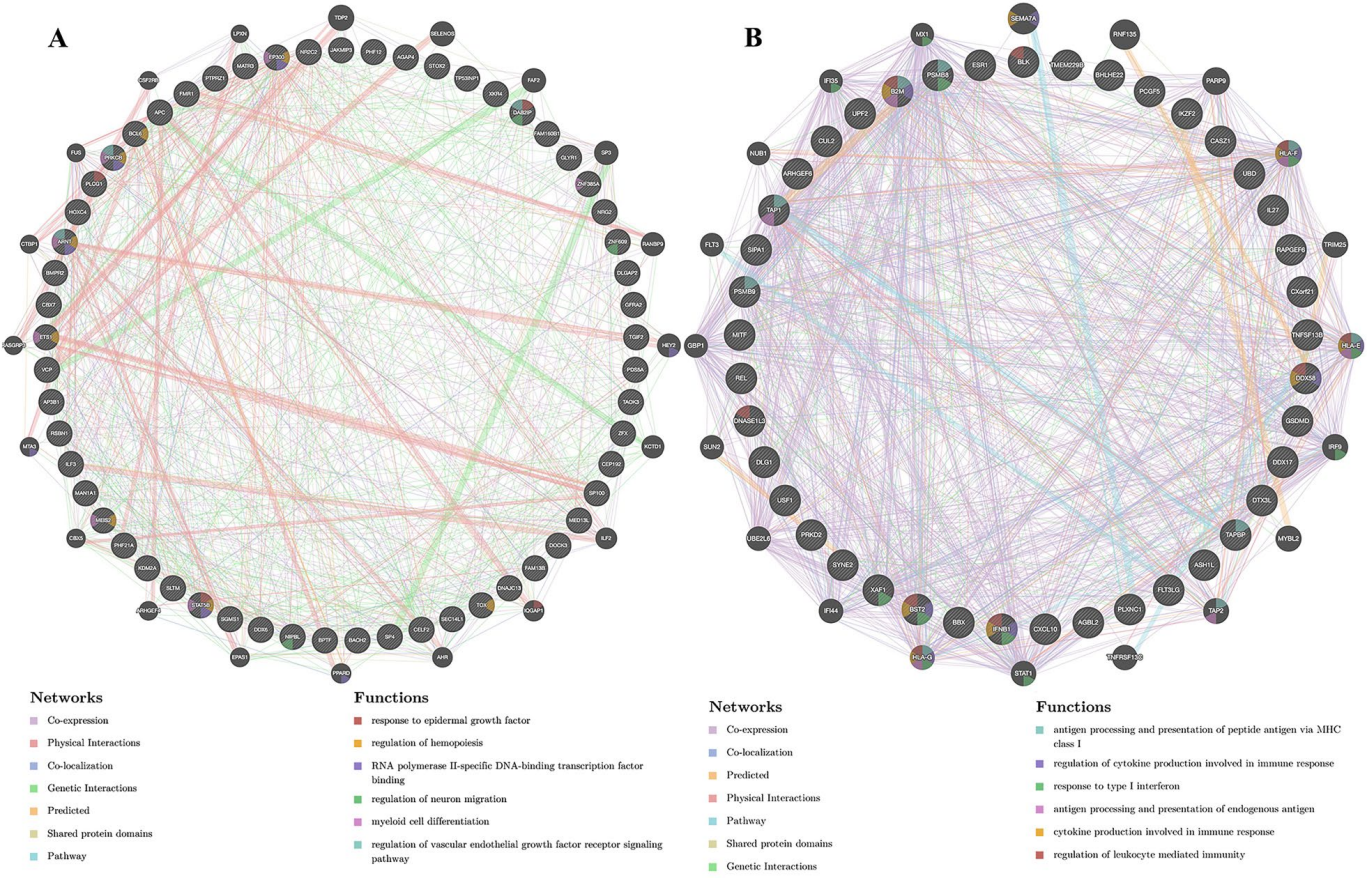
PPI network of target genes. PPI network constructed by miR-129-related genes (**A**) and IRF2-related genes (**B**). PPI, Protein–protein interaction

We then explored RFX5-related transcription factor targets and miRNA targets to further clarify the mechanism of STAD. The three most significant miRNA targets were MIR-129, MIR-19A/MIR-19B and MIR-507 (Table 2). The three most significant transcription factor targets were V$IRF2_01, V$PEA3_Q6, and V$ELF1_Q6 (Table 2). We also constructed a PPI network to explore the potential functions of miR-129 and IRF2. The data showed the miR-129 regulatory network was mainly responsible for response to epidermal growth factor, regulation of hemopoiesis, contractile fiber, myeloid cell differentiation, and neuron migration regulation (Fig. 6A). The IRF2 regulatory network was mainly responsible for regulation of cytokine production involved in immune response, response to type I interferon, and regulation of leukocyte mediated immunity (Fig. 6B).

**Table 2:** The miRNA and transcription factor target of RFX5 in STAD

Enriched category	Target	Edge number	*p*-value
miRNA target	GCAAAAA, MIR-129	57	0.004
	TTTGCAC, MIR-19A, MIR-19B	131	0.04
	GTGCAAA, MIR-507	45	0.04
Transcription factor target	V$IRF2_01	42	0
	V$PEA3_Q6	77	0
	V$ELF1_Q6	83	0

### Validation of the expression and prognosis value of RFX6 in STAD

**Fig. 7:**
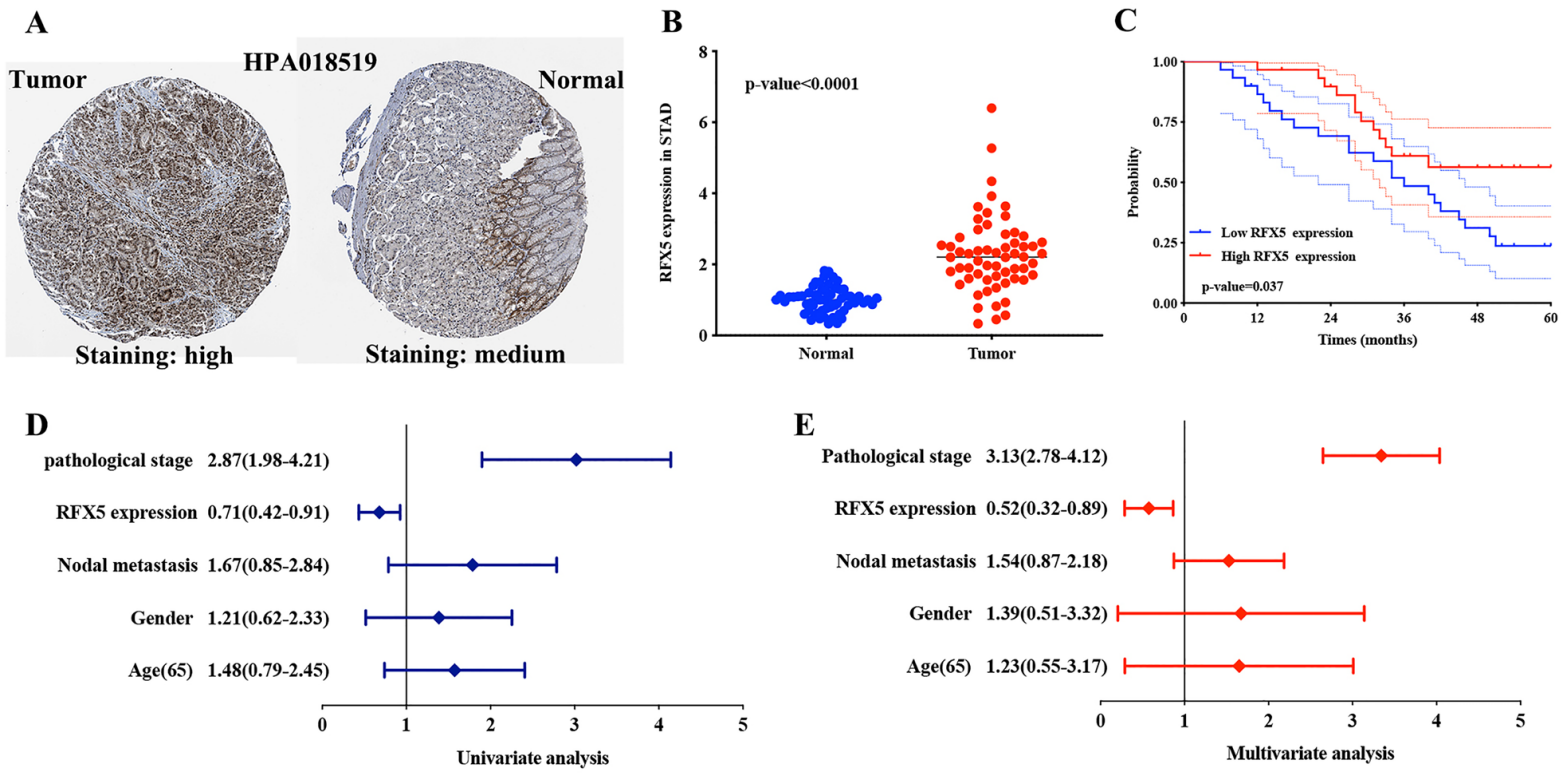
Verification of the expression and prognostic value of RFX5 in STAD. **A** The immunohistochemistry of RFX5 in STAD tissues and gastric tissues. **B**, **C** The mRNA level and prognostic value of RFX5 in STAD. **D**, **E** Univariate and multivariate cox regression analysis considering RFX5 expression and clinicopathologic features in STAD. STAD, stomach adenocarcinoma

The immumohistochemical staining revealed that RFX6 was high staining in STAD tissues while it was medium staining in normal tissues (Fig. 7A). The result of qRT-PCR indicated upregulation of RFX5 in STAD tissues versus normal tissues (Fig. 7B, *p* < 0.001). Moreover, prognosis analysis revealed that STAD patients with high RFX5 level had a better OS versus those with low RFX5 level (Fig. 7C, *p* = 0.037). Further univariate and multivariate analyses suggested that pathological stage and RFX5 could be a potential independent prognostic factor for STAD (Fig. 7D, E).

## Discussion

RFXs alterations have also been identified in various types of cancers, including diffuse large B cell lymphoma, acute myeloid leukemia [14]. RFXs exert a vital function in immune responses and are involved in the regulation of tumor cell growth and proliferation [7, 15–17]. Moreover, RFXs may act as prognostic biomarkers for types of cancer [7]. RFXs may also play a vital role in the development and prognosis in STAD. The study about the potential biological impact of RFXs in STAD is very limited and we performed this analysis.

From the overall perspective of RFXs, this study systematically analyzed the expression, prognostic value, immune infiltration, and related functions of RFXs in STAD. Expression analysis revealed that mRNA level of RFX1, RFX3, RFX4, RFX5, RFX7 and RFX8 was significantly elevated in STAD tissue versus adjacent normal tissue. Further prognostic analysis indicated that STAD patients with high RFX5 and RFX7 expression had a better overall survival, first progression, and post-progression survival. The data of qRT-PCR further verified our result. Univariate and multivariate analyses suggested that pathological stage and RFX5 could be a potential independent prognostic factor for STAD. Actually, many RFXs had been suggested as prognostic biomarkers for many types of cancer. RFX1 acted a prognostic biomarker in hepatocellular carcinoma and low RFX1 was correlated with poor prognosis [18]. Moreover, high expression of RFX4 is associated with tumor progression and poor prognosis in patients with glioblastoma [7]. Another study suggested RFX6, as a prognostic biomarker for melanoma [19].

Another vital finding of our study was that RFX5 expression was significantly associated with the abundance of immune cells, the expression of immune biomarkers and tumor mutational burden score in STAD. Immune cell infiltration plays an important role in tumor progression and metastasis and can affect patient prognosis in many ways [20]. Previous study revealed that the infiltration abundance of activated memory CD4 T cells and CD8 T cells had a significant effect on the overall survival of STAD patients [21]. Moreover, many immune-related biomarkers were suggested as prognostic biomarkers and therapy target of STAD, including PDCD1, and CTLA4 [22, 23].

In order to further clarify the potential mechanism of STAD, we then performed functional enrichment analysis and target analysis. Functional enrichment analysis revealed that RFX5 and its related genes were mainly involved in T cell activation, antigen receptor-mediated signaling pathway, cell adhesion molecules, and Th17 cell differentiation. We also identified certain miRNA and transcription factor targets of RFX5, including MIR-129, MIR-19A/MIR-19B, MIR-507, V$IRF2_01, V$PEA3_Q6, and V$ELF1_Q6. miR-129 was a novel therapeutic target and biomarker and inhibited tumor progression in STAD [24–26]. miR-507 could suppress the progression of STAD by regulating CBX4 and HIF-1α pathways [27]. Moreover, IRF2 serve as tumor suppressor and inhibit tumor progression in STAD [28, 29].

In conclusion, the above results suggest that RFX5 was a candidate prognostic biomarker and associated with immune infiltration in STAD. And it provides a theoretical basis for further study of the function and mechanism of RFX5 in STAD in the future.

## Supplementary Information

**Additional file 1: Figure S1**. The mRNA levels of RFXs in different pathological stage.

## Data Availability

The analyzed datasets generated during the study are available from the corresponding author on reasonable request.
