# Supplementary figures and images for: Identification of RFX5 as prognostic biomarker and associated with immune infiltration in stomach adenocarcinoma
Source: Eur J Med Res. 2022 Aug 31;27:164. doi: 10.1186/s40001-022-00794-w (PMC9429337; doi:10.1186/s40001-022-00794-w)

**Supplementary Fig 1. The mRNA levels of RFXs in different pathological stage.**


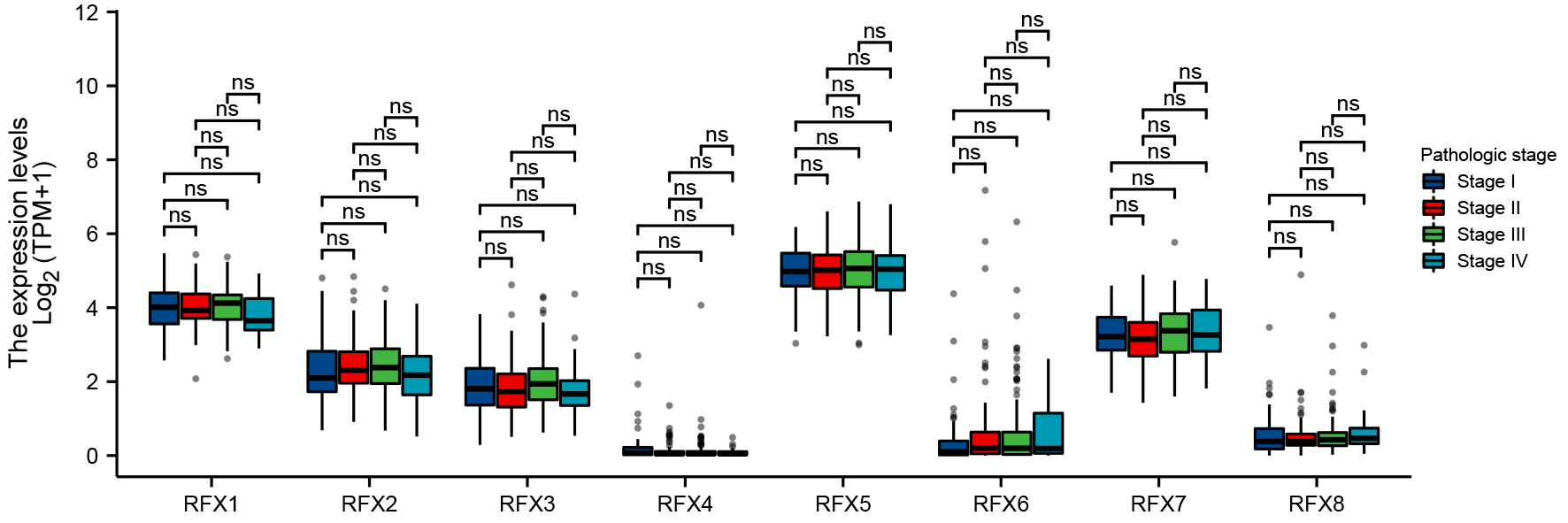

Supplement: Supplementary file 1 — Additional file 1: Figure S1. The mRNA levels of RFXs in different pathological stage. [file 40001_2022_794_MOESM1_ESM.docx]
